# Fracture Resistance of Posterior Tooth-Supported Cantilever Fixed Dental Prostheses of Different Zirconia Generations and Framework Thicknesses: An In Vitro Study

**DOI:** 10.3390/ma17010263

**Published:** 2024-01-04

**Authors:** Anna-Luisa Klotz, Janina Halfmann, Stefan Rues, Wolfgang Bömicke, Peter Rammelsberg, Andreas Zenthöfer

**Affiliations:** Department of Prosthodontics, Dental School, University of Heidelberg, Im Neuenheimer Feld 400, 69120 Heidelberg, Germany; janina.halfmann@hotmail.de (J.H.); stefan.rues@med.uni-heidelberg.de (S.R.); wolfgang.boemicke@med.uni-heidelberg.de (W.B.); peter.rammelsberg@med.uni-heidelberg.de (P.R.); andreas.zenthoefer@med.uni-heidelberg.de (A.Z.)

**Keywords:** cantilever fixed dental prosthesis, FDP, fracture load, zirconia, 3Y-TZP, 4Y-PSZ, 5Y-PSZ, gradient technology

## Abstract

The rehabilitation of free-end situations is a frequent indication in prosthetic dentistry. Cantilever fixed dental prostheses (cFDPs) made of 1st and 2nd generation zirconia are one treatment option. Due to a unique gradient technology, combinations of different zirconium dioxide generations are thus feasible in one restoration. However, data about these materials are rare. The purpose of this study was therefore to investigate the fracture resistance and fracture modes of tooth-supported cFDPs fabricated from different zirconia materials (gradient technology) and different framework thicknesses. A total of 40 cFDPs were fabricated using the CAD/CAM approach and belonged to five test groups. The different groups differed in the yttria content, the proportion of the tetragonal/cubic phases, or in wall thickness (0.7 mm or 1 mm). After completion, the cFDPs were subjected to thermal cycling and chewing simulation (1.2 × 10^6^ load cycles, 108 N load). Afterwards, cFDPs were statically loaded until fracture in a universal testing machine. A non-parametric ANOVA was compiled to determine the possible effects of group membership on fracture resistance. In addition, post-hoc Tukey tests were used for bivariate comparisons. The mean fracture loads under axial load application ranged from 288 to 577 N. ANOVA detected a significant impact of the used material on the fracture resistances (*p* < 0.001). Therefore, the use of cFDPs fabricated by gradient technology zirconia may not be unreservedly recommended for clinical use, whereas cFPDs made from 3Y-TZP exhibit fracture resistance above possible masticatory loads in the posterior region.

## 1. Introduction

The rehabilitation of unilateral or bilateral free-end situations is a frequent indication in prosthetic dentistry. While edentulous spaces neighbored by teeth can be restored with end-abutment tooth-supported fixed dental prosthesis (FDPs) if the distribution and the prognosis of the abutment teeth are adequate, removable dental prostheses are commonly indicated for free-end situations. However, alternatively, strategic implant insertion is required if FDPs are desired. If implants are not realizable for medical, anatomical, or financial reasons, the shortened dental arch can also be restored using cantilever fixed dental prosthesis (cFDPs) [1]. Albeit conventional cFDPs outperform removable dental prostheses with respect to complication and survival rates, as well as the resulting oral health-related quality of life, they fall behind end-abutment FDPs regarding complication and survival rates after 10 years of clinical use [2]. On closer inspection, cFDPs show more biological (e.g., loss of vitality, loosening of abutment teeth) and more technical (e.g., retention loss, veneer or framework fracture) complications [3,4]. Consequently, the annual loss rates for cFDPs are approximately twice as high as those for end-abutment FDPs, at some 2% [2]. For this reason, when planning cFDPs, a number of aspects should be considered. At least two, preferably vital teeth, should serve as abutments. Furthermore, a maximum of one extension pontic in premolar width and tangential gingival support should be realized. Dynamic occlusion contacts on the extension pontic should be avoided. Beside these aspects, the design (preparation and connector design) and the material used seem to have a crucial impact [5]. For the sake of completeness, it should be mentioned that strategic implants can serve as abutments for cFDPs, too. If, for example, one or two implants can be placed in the premolar region but not in the molar region due to reduced bone quantity and/or quality, implant-supported cFDPs can also be a viable treatment option. Overall survival rates are similar or even slightly more favorable to those seen for the tooth-supported variant; however, predominant complications are screw loosening, chipping, and decementations, especially if only one implant anchors the cFDP [6,7]. With regard to material selection, precious and non-precious metals (especially CoCr alloys) and zirconia have been recommended as framework materials for FDPs [8]. Especially zirconia is now being used more and more frequently due to its favorable mechanical and esthetic properties as well as its good biocompatibility. Zirconia enables a wide range of indications, and meanwhile, due to the introduction of highly translucent zirconia variants, monolithic processing [9]. Zirconia occurs in different solid phases, depending on the applied temperature. In this context, the tetragonal phase features favorable mechanical properties. For this reason, stabilizers such as CaO, MgO, or Y_2_O_3_ help stabilize zirconia in the tetragonal phase. First-generation zirconia (3Y-TZP) (3mol% yttria-stabilized tetragonal zirconia polycrystal) contains about 85% of the tetragonal phase, which results in high flexural strengths but low translucency (40%). Changes in the compositions of the different phases of zirconia have led to more translucent materials compared to 3rd generation zirconia (5Y-PSZ), which contains approximately 50% of both tetragonal and cubic phases and an yttria content of 5 mol% and therefore has improved optical appearance (translucency 49%) but lower flexural strengths [10,11,12]. Nevertheless, there is currently only clinical data on cFDPs made of 1st and 2nd generation zirconia (3Y-TZP) [13]. However, data from a laboratory study indicate that 3Y-, 4Y-, and 5Y-zirconia variants may be employed as materials for the fabrication of cFDPs [14]. In addition, monolithic restorations of different zirconium dioxide generations are now ready-to-use in one restoration due to multilayer technology. In this context, a distinction is made between polychromic multilayer and uniform compositions (Multilayer M3Y, M4Y, M5Y, and M6Y) and polychromic multilayer and hybrid compositions (M3Y and 5Y, M3Y and 4Y, M4Y and 5Y) [14]. These materials aim to combine favorable esthetics with good mechanical properties. However, in electron microscopy, 3Y-TZP demonstrated a higher grain consistency compared to the multilayer materials [15]. One producer of such materials is the dental company Ivoclar (Ivoclar Vivadent AG, Schaan, Liechtenstein), which offers IPS e.max ZirCAD Prime ((ML 3Y/5Y): Incisal: 5Y-PSZ; Dentin 3Y-TZP) and IPS e.max ZirCad Prime Esthetic ((ML 4Y/5Y): Incisal: 5Y-PSZ; Dentine 4Y-PSZ). Favorable material parameters are described for both materials by the manufacturer (ZCP: flexural strength: 650 MPa (incisal); 1200 MPa (dentin); fracture toughness: >5.0 MPa∙m^0.5^; ZCPE: flexural strength: 650 MPa (incisal); 850 MPa (dentin); fracture toughness: 3.6 MPa∙m^0.5^), which permit a wide range of therapy indications [16].

Irrespective of the used zirconia, suitable framework dimensions—adapted according to the planned type and location of FDPs—are essential for long-term survival. Depending on the manufacturers’ instructions and the zirconia type used, the minimum connector size is specified as 9 mm^2^ in the anterior region and 12 mm^2^ in the posterior region (3Y-TZP), while the manufacturers’ recommendations hardly vary, especially for the different zirconia generations. When looking at thematic literature, the adequate connector height can reduce the stress concentration [17]. Furthermore, it has been demonstrated that the typical failure pattern of cFDPs is a fracture at the distal wall located close to the extension pontic. For this reason, the wall thickness between the posterior anchoring part and the cantilever of the cFDP seems to be crucial with respect to fracture resistance [15,18,19].

The purpose of this study was therefore to investigate the performance of tooth-supported cantilever fixed dental prostheses fabricated from different zirconia materials (gradient technology) and different frameworks.

## 2. Materials and Methods

### 2.1. Study Design

In this in vitro study, 40 cFDPs were examined in five groups (n = 8/group). In four test groups, cFDPs with wall thicknesses of either 0.7 mm or 1.0 mm were milled from two different multilayer blanks. The multilayer blanks consist of a 3 mm-thick enamel layer, a 4 mm-thick transition layer, and a dentine layer forming the rest of the blank. While the first multilayer blank type (ML 3Y/5Y; IPS e.max ZirCAD Prime, Ivoclar Vivadent AG), combined 3Y-TZP (dentine) and 5Y-PSZ (enamel), the second blank type (ML 4Y/5Y; IPS e.max ZirCAD Prime Esthetic, Ivoclar Vivadent AG) combined 4Y-PSZ (dentine) and 5Y-PSZ (enamel). In the fifth test group, cFDPs were milled from 3Y-TZP (3Y; IPS e.max ZirCAD LT, Ivoclar Vivadent AG) with a 0.7 mm wall thickness as a reference. An overview of the test groups is also presented in Table 1. The properties of the different zirconia types used with the three different blank types are listed in Table 2.

**Figure 1:**
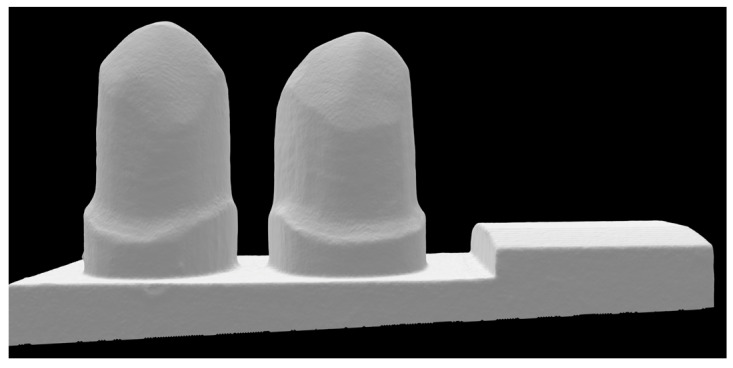
View of the simulated clinical setting with a missing mandibular first molar. The first and second premolars served as abutment teeth (44 and 45, according to FDI notation, 8 mm distance between the abutment tooth axes).

A clinical setting with a missing mandibular first molar was simulated. The first and second premolars served as abutment teeth (44 and 45 according to FDI notation, 8 mm distance between the abutment tooth axes) and were prepared on a typodont model using a chamfer design (ANA-4, Frasaco, Tettnang, Germany). The preparation followed the general guidelines for ceramic restorations (see Figure 1) [20,21]. In terms of standardization, the plastic teeth were prepared with a laboratory handpiece fixed in the paralleling device (Degussa milling unit F1, Degussa: GB Dental und Goldhalbzeug, Frankfurt am Main, Germany) and diamond burs (356 040M HKP, NTI-Kahla GmbH Rotary Dental Instruments, Kahla, Germany). The prepared abutments had a unilateral preparation angle of 3°.

### 2.2. CFDP Design and Fabrication

**Figure 2:**
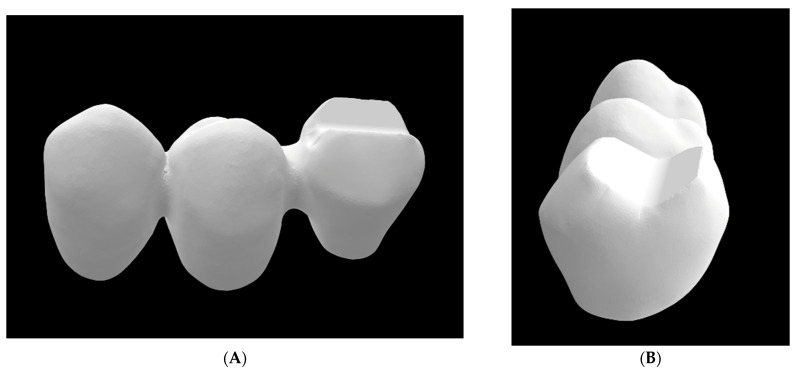
Different views of the cFDP show (**A**) the connector design and (**B**) the modified occlusal surface of the pontic.

**Figure 3:**
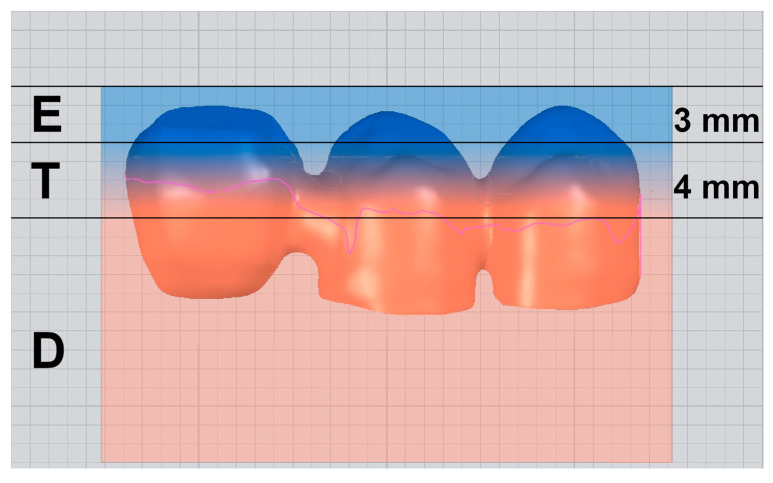
Nesting position of the cFDPs in the multilayer zirconia blanks, consisting of a 3 mm high enamel layer (E), a 4 mm high transition layer (T), and a dentine layer (D) forming the rest of the blank height. According to the manufacturer’s instructions, cFDP was positioned 1 mm from the upper surface.

The prepared teeth were digitized to obtain a virtual 3D model using a laboratory laser scanner (D2000; 3Shape; Copenhagen, Denmark), and the cFDP with 0.7 mm wall thickness was virtually designed on the computer using dental design software (DentalDesigner Build Ver. 2018.2.0; 3Shape) with a 20 µm marginal gap width and a 60 µm cement gap width. The minimum connector cross section exceeded 12 mm^2^ area and measured about 4 mm in height and 3 mm in width. The pontic was designed to be pontiform, that is, basally rounded (see Figure 2A,B). After exporting the cFDP geometry, the cusps of the pontic in the distal area were modified to be planar with an exact 30° tilt with respect to the horizontal plane (Geomagic DesignX Build Ver. 2022.0: 3D Systems; Mörfelden-Walldorf, Germany), which enabled a standardized force application in artificial aging and fracture load tests. For the cFDP with 1.0 mm wall thickness, the previous job with 0.7 mm wall thickness was copied, and the wall thickness was modified to 1.0 mm. After stl-export of the cFDP geometry with 1.0 mm wall thickness, the connector and pontic were deleted and replaced by the connector and pontic of the cFDP with 0.7 mm thickness in a position shifted by 0.3 mm in the distal and occlusal directions. The nesting of the cFDPs followed the manufacturer’s recommendations; restorations were suited 1 mm away from the upper surface of the blanks; the cFDPs cusps were located in the enamel layer; and the upper half of the connector was located in the transition layer (see Figure 3). All cFDPs were then milled using a 5-axis milling machine (PM7; Ivoclar Vivadent) and sintered (Programat S1; Ivoclar Vivadent) according to the manufacturer’s instructions.

### 2.3. Production of the Cobalt-Chromium Alloy Tooth Analogs

**Figure 4:**
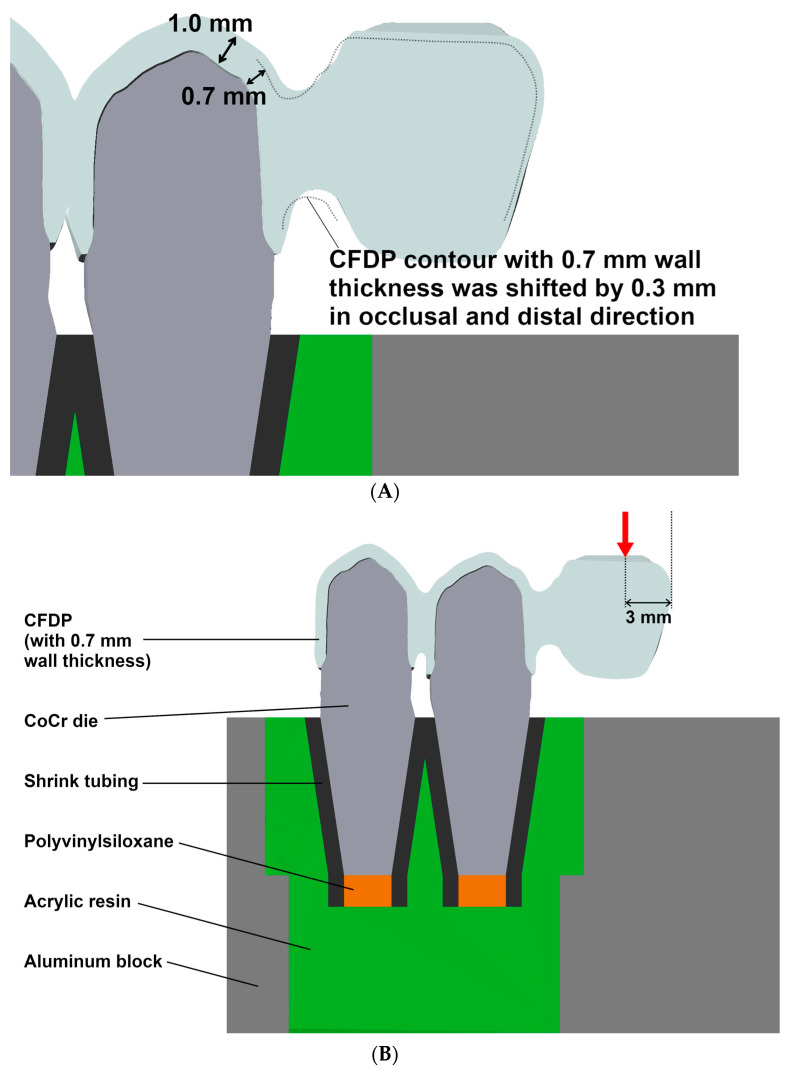
(**A**) Design of the pontic and connector of the cFDP with a 1.0 mm wall thickness based on the geometry of the cFDP with a 0.7 mm wall thickness; (**B**) test setup and load application (red arrow) of the cFDP cemented to resiliently embedded metal stumps.

Metal dies made of a cobalt-chromium alloy (Remanium Star; Dentaurum GmbH & Co. KG; Ispringen, Germany) were used as abutments for the cFDPs. Therefore, the digitally prepared tooth geometries were complemented by standardized conical roots with a rectangular cross section and 10 mm length. The analogs were 3D-printed (Max UV; Asiga, Erfurt, Germany) using a combustible resin (V-Print Cast; Voco GmbH, Cuxhaven, Germany). The printed dies were then embedded (One-micro-Plus; feguramed GmbH) in a muffle with a silicone ring and a casting mold made from combustible resin. Finally, the metal dies were cast (iQ vacuum pressure caster; Kulzer GmbH, Hanau, Germany) with cobalt-chromium alloy. After devestment, the metal teeth were sandblasted using a pressure of 0.2 MPa with 50 µm alumina particles. To simulate tooth mobility, a shrink tubing was placed over the root portion of the cobalt-chromium teeth, cut off 2 mm below the apical end of the root, and the apical opening of the shrink tubing was filled with polyvinylsiloxane (Flexitime Correct Flow, Kulzer GmbH). Thus, an abutment tooth resilience of about 1 µm/N in the vertical direction and 3–4 µm/N in the horizontal direction was simulated, which is in the range of clinical tooth resilience (see Figure 4A).

### 2.4. Fabrication of the Typodont Replicas

A block-shaped mold with the negative shape of the occlusal cFDP geometry was planned in the CAD software (Geomagic DesignX Build Ver. 2022.0, 3D Systems). The insertion direction of the cFDPs (identical to the tooth axis directions) was oriented vertically. This mold was 3D-printed (V-Print Model, Voco GmbH, Cuxhaven, Germany) and attached with its planar upper side to a paralleling device. Aluminum blocks received two drillings, each 14 mm in diameter and 8 mm in distance. After provisionally joining metal dies and cFDPs, cFDPs were attached to the paralleling device via the mold, such that the vertical direction was identical to the insertion direction, and the die roots were covered with shrink tubing and lowered into the drillings of the aluminum block and embedded with acrylic resin (Technovit 4071; Kulzer GmbH).

### 2.5. Cementation

After the removal of each provisionally attached cFDP, the fit of the cFDP on the typodont replica was checked again. If the fit was inadequate, the embedding process for the dies had to be repeated. Before cementation, the inner crown surfaces and the prepared tooth surfaces were sandblasted (50 µm alumina particles, 0.1 MPa). Cementation was carried out with a glass ionomer cement (Ketac Cem, 3M Oralcare; Neuss, Germany). For this purpose, the aluminum block with metal dies was fixed in a universal testing machine (Z005, Zwick/Roell, Ulm, Germany). After application of the cement to the inner part of the cFDP anchors and manual placement of the cFDPs on the typodont replica, the cFDPs were axially loaded between the abutment teeth axes. After reaching a preload of 5 N, the load was increased by 10 N/s up to 190 N and by 1 N/s until the final load of 200 N was reached. Then, the load was held at 200 N for 6 min (complete hardening of the cement).

### 2.6. Artificial Aging/Chewing Simulation

The cFDPs were then stored for 24 h at 100% humidity and 37 °C in the heating cabinet (Heraeus Function Line). It was checked that no damage, such as fractures or cracks, occurred during cementation using a digital microscope (Smartzoom 5, Carl Zeiss AG, Jena, Germany). The cFDPs were then stored in deionized water at 37 °C for 3 days in a heating cabinet (Heraeus Function Line). Then, all cFDPs were subjected to 10,000 thermal cycles and water bath temperatures of 6.5 °C and 60 °C (Thermocycler TC 1; SD Mechatronik GmbH, Feldkirchen-Westerham, Germany). The dwelling time in the baths lasted 45 s each, with 2 s of drip time and a transfer time of 5 s. After thermocycling, samples were visually inspected again for damage, such as cracks within the cFDPs or decementation.

**Figure 5:**
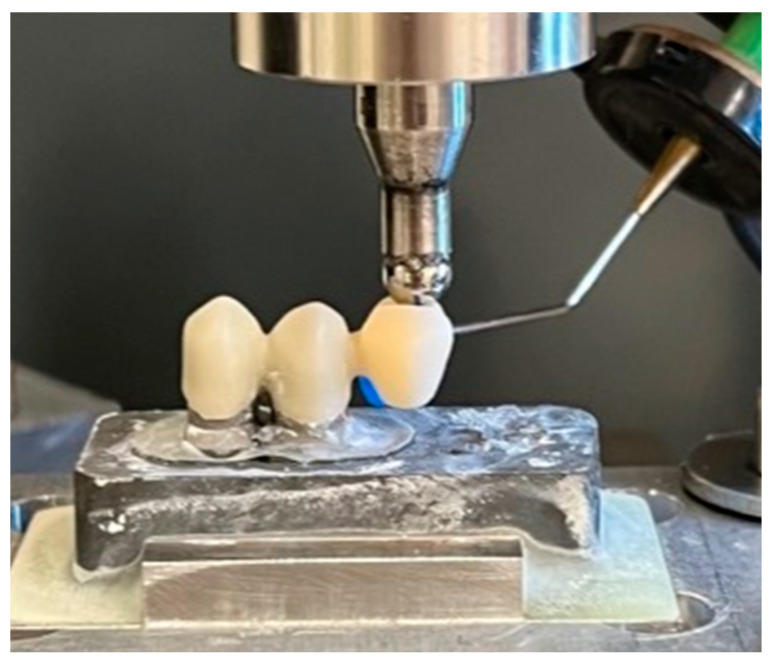
Picture taken during the fracture tests of the cFDPs in the universal-testing machine with a sensor enabling body-borne sound signals.

In the next step, cFDPs were subjected to cyclic mechanical loading (1.2 × 10^6^ load cycles), as recommended in several studies [22,23,24], in a chewing simulator (Chewing Simulator CS-4.8, SD Mechatronik GmbH) immersed in deionized water. The force was transmitted by a piston in the form of a stainless steel ball (Ø 6 mm) connected to a mass of 9 kg. By using a spring-damper system, force magnitudes of 108 N were generated at a lowering speed of the crosshead of 30 mm/s during the chewing simulation; the static force after the impact was 90 N. The cFDPs were adjusted in the chewing simulator such that the metal ball came to rest evenly on both cusps at a distance of 3 mm from the distal end (see Figure 4B and Figure 5). This position was previously marked on the cFDPs by a line drawn and checked under a digital microscope (Smartzoom 5). The mechanical loading of the pontic was performed axially as a pure vertical movement. Samples were regularly monitored during the chewing simulation, and any damaging events were noted in a protocol. In the event of a failure of a cFDP during chewing simulation, a fracture resistance of 108 N was assigned to the specimen.

### 2.7. Fracture Load Test

Samples surviving the chewing simulation were subjected to fracture load testing in a universal testing device (Z005, Zwick/Roell) with a crosshead speed of 0.5 mm/min. A hardened steel antagonist with a spherical shape (Ø 6 mm) was used. Load application position and load orientation were identical to the settings used during the chewing simulation. To detect and identify possible damage occurring before the final fracture, body-borne sound signals and videos were recorded during the fracture tests. The fracture tests ended when the test force dropped by more than 80% of the actual maximum test force. The maximum force recorded during each test was defined as the fracture resistance of the respective cFDP.

### 2.8. Statistical Analysis

For each test group, mean values and standard deviations were computed for the recorded fracture resistances. In addition, the outcome was visualized using box plot diagrams. A non-parametric analysis of variance (ANOVA) was performed after assigning ranks that increased first with an increase in completed chewing cycles and second with an increase in fracture resistance. Test groups differed in the factors “blank type” and “wall thickness”. Pairwise post-hoc comparisons were conducted using Tukey tests. All analyses were performed with a significance level of α = 0.05 with the aid of SPSS Ver. 28 (IBM; Armok, NY, USA).

## 3. Results

### 3.1. Artificial Aging

For none of the cFDPs, any damage (cracks within the zirconia, decementation) was found during inspection after thermocycling. During the chewing simulation, four samples failed (see Table 3). Only the groups 3Y-0.7 and ML 3Y/5Y-0.7 showed no failure during chewing simulation. The earliest failure during artificial aging occurred in group ML 4Y/5Y-0.7 after only 3000 cycles; all other failures occurred before 300,000 cycles were reached. The localization of the fracture during the chewing simulation was the distal wall at the connector area.

### 3.2. Fracture Resistance

**Figure 6:**
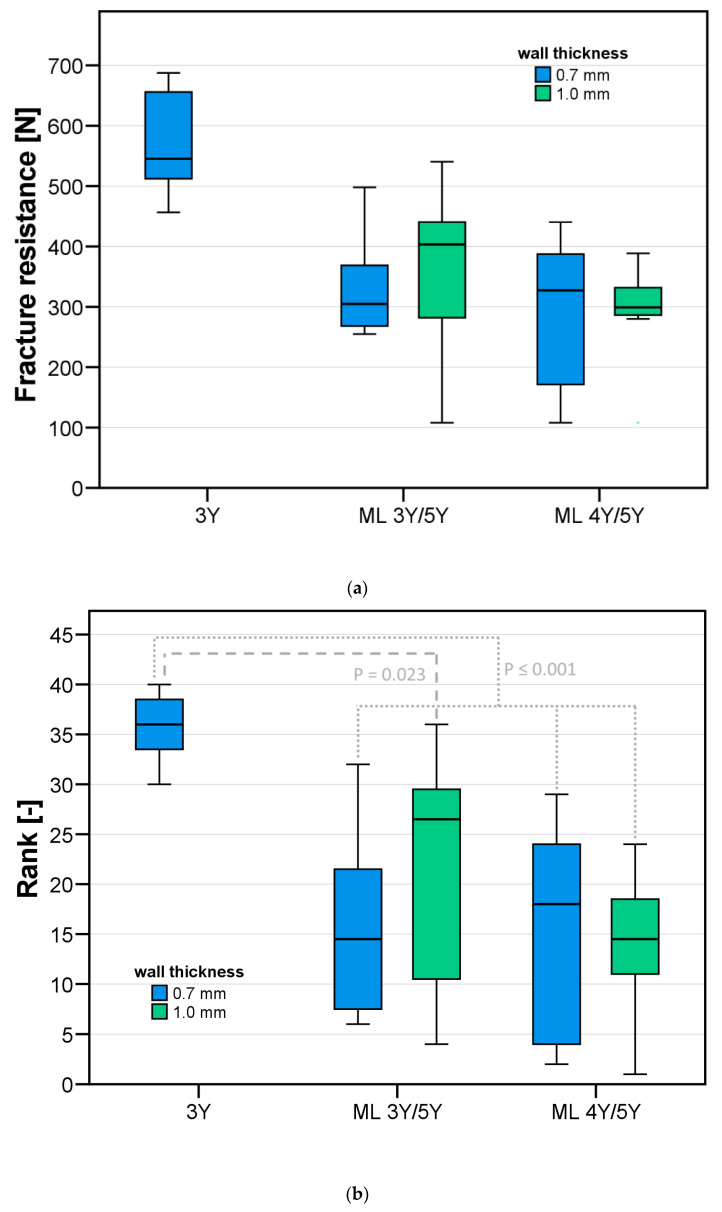
Results of the fracture load test of the different test series: (**a**) fracture resistance and (**b**) ranks according to failure during aging and respective fracture resistance. *p*-values were calculated by unifactorial ANOVA.

During the fracture load test, a complete fracture of the cFDP was the only major event. In this context, the force and sound signals were recorded, and the test was visually monitored. All results are summarized in Table 3 and displayed in Figure 6a,b. The mean fracture load under axial load application was similar for all cFDPs fabricated from multilayer zirconia, ranging between 290 N and 363 N. In contrast, with a mean fracture resistance of 577 N, cFDPs with a 0.7 mm wall thickness made from 3Y-TZP were able to withstand almost twice as much maximum test force. The same effects could be seen when looking at the ranks assigned to the samples.

A non-parametric ANOVA detected a significant impact of the blank type on sample rank (*p* < 0.001), whereas no association between wall thickness and rank could be detected (*p* = 0.452). On closer inspection, pairwise post-hoc tests indicated that the test group 3Y-0.7 significantly differed from all other test groups (*p* ≤ 0.023 for all tests), whereas pairwise comparisons between all cFDP groups fabricated from ML zirconia were not significant (*p* ≥ 0.449) (see Table 4).

**Figure 7:**
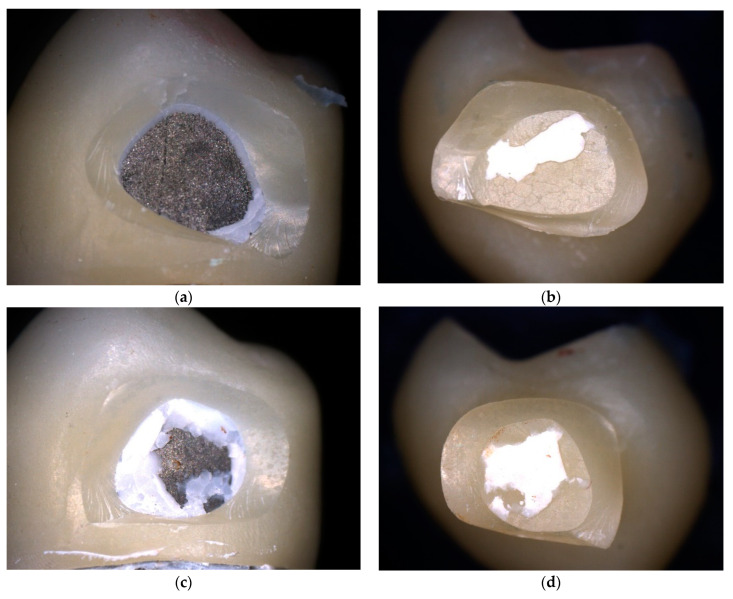
Localization of the fractures of the cFDPs (**a**–**d**): The localization of the fracture was the distal wall at the connector area. (**a**,**b**) as well as (**c**,**d**) each show one sample, abutment-sided and pontic-sided.

With regard to fracture modes, four cFDPs fractured between the abutment teeth. The other cFDPs fractured at the distal wall close to the connector area (see Figure 7a–d).

## 4. Discussion

All frameworks were fabricated using the CAD/CAM process. A particular advantage of this procedure was that all cFDPs could be produced in a standardized manner. By using standardized milling parameters, industrially produced blanks, and similar positioning, there were no differences in material dimensions. However, studies found that the sintering process and positioning of restorations within a multilayer zirconia blank have a minor effect on the mechanical properties of the prostheses [25].

No problems were encountered in the design of the cFDPs when using the standard settings of the design software (Dental Designer Build Ver. 2018.2.0; 3Shape). With regard to the design of the cFDPs, the dimension of the connector is decisive for the ultimate load of the FDPs [26]. However, an increase in the connector cross-section only causes an increase in the loads needed to fracture FDPs. In the present study, a connector cross-section of at least 12 mm^2^ (4 mm × 3 mm) was selected for the cFDPs framework, as recommended by the manufacturer. Bahgat et al. proved in their study that a connector of 4 mm × 3 mm is sufficient to withstand masticatory forces in the posterior region over a long period of time [27]. Since none of the cFDPs fractured across the connector area, an increase in the connector cross-section of ML 4Y/5Y and ML 3Y/5Y probably would not have led to another outcome in this specific study. In reconciliation with clinical reality, one might also look at the available space, guaranteeing the patient’s hygiene ability, which is frequently limited.

When looking at this study setting, previous studies have demonstrated that three-unit FDPs had a significantly higher fracture load on metal abutments than on resin abutments [28]. In addition to the elastic modulus of the abutment material used, the relevant stiffness of the restorations also depends on other factors, such as the bearing of the dies. Metal abutment teeth with simulated natural resilience have proven to provide a good correlation between results from in vitro and clinical testing [26,27,28,29,30]. Therefore, in the present study, abutment tooth analogs made of a CoCr alloy were used. Furthermore, in vitro studies showed that resilient mounting of abutment teeth in the test model led to a decrease in fracture loads compared to firmly anchored teeth, especially after aging (thermocycling and chewing simulation) and subsequent destructive testing [28,29,30,31]. This is therefore more similar to clinical behavior and was therefore used in this study setting.

Any type of dental prosthesis must withstand the natural, regular chewing load. In this study, the chewing load was simulated in a chewing simulator with 1.2 million loading cycles at a force amplitude of 108 N. The load was applied to the cFDPs in a chewing simulator. Krejci et al. describe that 240,000 cycles correspond to an intraoral wear time of one year (658 cycles per day) [22]. Similar values were also documented in studies by De Long et al. and Sakaguchi et al., with approximately 250,000 loading cycles per year [23,24]. Accordingly, the 1.2 million loading cycles in this study represent an intraoral wearing time of the denture of approximately 5 years. According to Schindler et al., physiological masticatory forces of 20–120 N are applied to the teeth during mastication [32]. In their studies, Lundgren and Laurell found that the average masticatory load on extension pontics was 50–150 N [33]. The 108 N chosen for the present study is thus approximately in the middle of the values used in other studies. However, the majority used force amplitudes that were much lower compared to this study [11,29,30,31,32,33]. Therefore, it can be assumed that a period longer than 5 years was simulated in the present study.

The fracture loads of all-ceramic restorations vary depending on the direction of load application. In this study, the load was applied axially to the cFDPs with a steel ball (diameter of 6 mm). Wolfart et al. described that an axial loading in the fracture test emphasizes the characteristic properties of zirconia [34]. For this reason, this load application was chosen. In this study setting, the mean fracture load ranged from 288 N (ML 4Y/5Y) to 577 N (ML 3Y/5Y). In comparison, Schmidt et al. tested cFDPs fabricated by different zirconia generations. The groups with artificial aging showed similar fracture values to those of this study (3Y-TZP: about 650 N; 4Y-PSZ: about 650 N; 5Y-PSZ: about 350 N) [11]. While examining the literature, Körber et al. found that the average maximum masticatory force for cFDPs in the posterior region is approximately 300 N [35]. Lundgren and Laurell found that the masticatory force for cFDPs with a shortened dental arch is much lower. In this context, the mean chewing force was measured to be 50 N and the maximum chewing force to be about 150 N [29]. Körber additionally determined a so-called safety factor of 200 N for cFDPs, which should be applied regardless of the maximum load [35]. The minimum fracture loads for cFDPs can therefore be set to 350 N. However, in this study, similar values were only seen for ZirCad LT and ZirCad Prime with a wall thickness of 1 mm. The other tested materials and the wall thickness of 0.7 mm fell behind these values. One reason could have been that high tensile stresses occur on the occlusal surface with cFDPs where the restoration was placed in the enamel and transition layers of the multilayer blanks (Figure 3). These layers contain 3–5% Y_2_O_3_, resulting in a stabilization of the cubic phase next to the tetragonal phase. The cubic phase provides improved translucency but lower flexural strength and fracture toughness due to a lack of transformation toughening compared with the tetragonal phase [36]. Furthermore, the higher grain inconsistency in the multilayer materials might be a risk factor for crack formation and, therefore, reduced fracture loads [15].

An in vitro study by Guazzato et al. showed that the ultimate load of the overall structure depends on the material at the bottom of the workpiece, i.e., the tensile side [37]. In this view, most studies refer to end-abutment FDPs, whereas for cFDPs, the highest tensile stress at the pontic is at the top side of the connector [38,39,40]. As in gradient technology, the weaker material is on the top side to achieve higher levels of esthetic; these materials could be more susceptible to fracture as immediate failure of the entire structure occurs when the critical load of the material on the top side is reached. This could explain the comparatively poor fracture load values of the cFDPs using gradient technology.

However, in the clinical setting, the performance of cFDPs is also influenced by various other parameters, such as the configuration of the abutment teeth or the mode and type of cementation. In this context, the vitality of the abutment teeth seems to have a high impact on severe complications and survival [1]. Another important aspect is the type of cement used. For glass-ceramic restorations, the best fracture strength is described for adhesive cements [41]. For high-strength ceramic restorations, an increase in flexural strength after luting is controversially discussed [42], and the evidence on the exact influence of the cementation medium on clinical performance is limited [43]. When it comes to zirconia luting, multiple conditioning steps have to be performed for the abutment teeth and the restorations. These steps are time-consuming, technique-sensitive, and susceptible to contamination; consequently, clinicians frequently prefer conventional cementation with zinc-phosphate, glass ionomer, or resin-modified glass ionomer cements if conventional full-coverage FDPs should be placed. Clinical long-term studies indicate that zirconia full-coverage crowns and FDPs exhibit favorable long-term survival rates when conventional cements are used; decementations are rare [43]. To draw a clinically realistic picture, conventional cement was used in this study. However, analyzing the impact of luting cFDPs might be an interesting research theme and should be addressed in further studies. To this end, it should also be mentioned that implants can serve as abutments for cFDPs, too. Similar guidelines as for tooth-supported cFDPs, such as the premolar width of the extension pontic and material thicknesses, must be adhered to in order to achieve a good long-term prognosis. Even though implant-supported cFDPs with one anchoring implant are possible, complications seem to be more frequent [6,7]. To date, there is a lack of reliable, systematic, and long-term studies on the latter type of restoration.

### Limitations

However, when interpreting the study results, it should be kept in mind that the results were tested in an in vitro design. Laboratory studies cannot be unreservedly extrapolated to a possible clinical outcome [44], as laboratory tests give only a limited impression of the expected properties of the tested materials [45]. The human masticatory forces, occlusion, surrounding tissues, and food type and texture are all aspects that might influence the clinical performance of cFDPs [35,38].

One further limitation is the rather small sample size of the different test groups. However, the required sample size was calculated using an a priori power calculation: the mean difference according to strengths between 3Y-TZP and 5Y-PSZ (1000 MPa to 650 MPa) was 35%, and the typical coefficient of variation was 20%. For this reason, the required sample size was n = 7. For safety and planning reasons (eight chambers in the chewing simulator), the number of cFDPs was increased to eight.

## 5. Conclusions

Within the limitations of this laboratory study, the use of cFDPs fabricated by gradient technology (4Y-PSZ or 5-PSZ) may not be unreservedly recommended for clinical use. However, fracture loads of cFDPs made from 3Y-TZP exceeded fracture loads of possible masticatory forces in the posterior region and can therefore be a suitable treatment option.

## Figures and Tables

**Table 1:** Details of the test groups.

Test Group	Sample Size	Zirconia Blank Type	Wall Thickness
3Y-0.7	N = 8	IPS e.max ZirCAD LT	0.7 mm
ML 3Y/5Y-0.7	N = 8	IPS e.max ZirCAD Prime	0.7 mm
ML 4Y/5Y-0.7	N = 8	IPS e.max ZirCad Prime Esthetic	0.7 mm
ML 3Y/5Y-1.0	N = 8	IPS e.max ZirCAD Prime	1.0 mm
ML 4Y/5Y-1.0	N = 8	IPS e.max ZirCad Prime Esthetic	1.0 mm

**Table 2:** Parameters provided by the manufacturer for the base materials of the zirconia blanks.

Zirconia Type	Phase Composition [%]		Material Parameters	
	Tetr.	Cubic	Flexural Strength [MPa]	Fracture Toughness [MPa m^0.5^]
3Y-TZP	100	0	1000 ± 200	5.00 ± 0.25
4Y-PSZ	75	25	850 ± 100	3.75 ± 0.25
5Y-PSZ	50	50	650 ± 50	3.75 ± 0.25

**Table 3:** Results of the artificial aging and fracture load tests for the different test series.

Test Group	Number of Failures during Chewing Simulation	Fracture Resistance [N]		Rank [22,23,24]	
		Mean Value	Standard Deviation	Mean Value	Standard Deviation
3Y-0.7	-	571	85	35.8	3.4
ML 3Y/5Y-0.7	-	330	82	15.6	9.1
ML 3Y/5Y-1.0	1	363	134	15.4	10.7
ML 4Y/5Y-0.7	2	290	128	21.6	11.6
ML 4Y/5Y-1.0	1	291	82	14.1	6.9

**Table 4:** Results of the non-parametric 2-way ANOVA.

Factor/Factor Combination	F	*p*-Value
Blank type	14.768	<0.001
Wall thickness	0.578	0.452
Blank type * Wall thickness	1.346	0.254

## Data Availability

The data that support the findings of this study are available from the corresponding author (AK) upon reasonable request.
